# Let-7b Inhibits Human Cancer Phenotype by Targeting Cytochrome P450 Epoxygenase 2J2

**DOI:** 10.1371/journal.pone.0039197

**Published:** 2012-06-25

**Authors:** Fuqiong Chen, Chen Chen, Shenglan Yang, Wei Gong, Yan Wang, Katherine Cianflone, Jiarong Tang, Dao Wen Wang

**Affiliations:** 1 Departments of Internal Medicine and Gene Therapy Center, Tongji Hospital, Tongji Medical College, Huazhong University of Science and Technology, Wuhan, People's Republic of China; 2 Centre de Recherche, Institut Universitaire de Cardiologie et de Pneumologie de Québec, Université Laval, Québec City, Québec, Canada; Northwestern University, United States of America

## Abstract

**Background:**

MicroRNAs (miRNAs) are small, noncoding RNA molecules of 20 to 22 nucleotides that regulate gene expression by binding to their 3′ untranslated region (3′UTR). Increasing data implicate altered miRNA participation in the progress of cancer. We previously reported that CYP2J2 epoxygenase promotes human cancer phenotypes. But whether and how CYP2J2 is regulated by miRNA is not understood.

**Methods and Results:**

Using bioinformatics analysis, we found potential target sites for miRNA let-7b in 3′UTR of human CYP2J2. Luciferase and western blot assays revealed that CYP2J2 was regulated by let-7b. In addition, let-7b decreased the enzymatic activity of endogenous CYP2J2. Furthermore, let-7b may diminish cell proliferation and promote cell apoptosis of tumor cells via posttranscriptional repression of CYP2J2. Tumor xenografts were induced in nude mice by subcutaneous injection of MDA-MB-435 cells. The let-7b expression vector, pSilencer-let-7b, was injected through tail vein every 3 weeks. Let-7b significantly inhibited the tumor phenotype by targeting CYP2J2. Moreover, quantitative real-time polymerase chain reaction and western blotting were used to determine the expression levels of let-7b and *CYP2J2* protein from 18 matched lung squamous cell cancer and adjacent normal lung tissues; the expression level of CYP2J2 was inversely proportional to that of let-7b.

**Conclusions:**

Our results demonstrated that the decreased expression of let-7b could lead to the high expression of CYP2J2 protein in cancerous tissues. These findings suggest that miRNA let-7b reduces CYP2J2 expression, which may contribute to inhibiting tumor phenotypes.

## Introduction

Human cytochrome P450 (CYP) epoxygenase, CYP2J2, catalyzes the epoxidation of arachidonic acid into four regioisomers of cis-epoxyeicosatrienoic acid (5,6-EET; 8,9-EET; 11,12-EET; and 14,15-EET) [1]. This enzyme seems to be primarily expressed in heart and vessel endothelial cells [2], and it has also been found in a variety of tissues including liver, lung, kidney, and gastrointestinal tissues [3]. Because differences in the catalytic efficiency of individual P450 isoforms results in different EET profiles for each [4], 11,12- and 14,15-EETs are the primarily arachidonic acid metabolites produced in various cells and tissues [5], [6].

Several studies have reported that EETs have diverse biological effects within the cardiovascular system. The EETs released from the endothelium activated calcium-sensitive potassium channels and resulted in hyperpolarization of smooth muscle cells and vascular relaxation [7]. Moreover, physiological concentrations of EETs or overexpression of *CYP2J2* reduced vascular cell adhesion molecule-1 (VCAM-1) expression and prevented leukocyte adhesion to the vascular wall [8]. The inhibitory effects of EETs show that they have anti-inflammatory effects in vascular system independent of their membrane-hyperpolarizing effects [8]. In addition, EETs also promoted endothelial cell proliferation, migration, and angiogenesis by activating both mitogen-activated protein kinase (MAPK) and phosphatidylinositol-3 (PI3)-kinase/Akt pathways [9].

On the other hand, other evidences indicate that epoxygenase overexpression or EETs treatment have potentially deleterious effects. In recent publications, CYP2J2-derived EETs were found to play important roles in a host of processes related to cancer cell behavior and tumor pathogenesis. We found high expression of CYP2J2 in human tumors, as well as in eight human-derived carcinoma cell lines, but not in adjacent normal tissues and nontumoral human cell lines [10]. Overexpression of CYP2J2 or addition of exogenous EETs markedly accelerated proliferation and metastasis of cancer cells in vitro and in vivo [10], [11]. CYP2J2 overexpression or addition of exogenous EETs protected human carcinoma cells from apoptosis by upregulating the antiapoptotic proteins, Bcl-2 and Bcl-xL, and by downregulating the proapoptotic protein, Bax [10]. In contrast, selective inhibitors of CYP2J2 had significant antitumor effects in vitro and in vivo and were associated with reduced EET biosynthesis [12]. Collectively, all the findings demonstrated the important and previously unrecognized roles of CYP2J2 and its EET products in carcinogenesis.

Increasing evidences indicate that miRNAs play important roles in diverse biological processes, such as proliferation, differentiation, and apoptosis during development [13], [14], [15]. Several studies have indeed described the aberrant expression in human tumors of miRNAs and their function controlling the expression of certain oncogenes and tumor suppressor genes [16], [17], [18]. For example, miR-15a and miR-16 are frequently deleted or downregulated in squamous cell carcinomas and adenocarcinomas of the lung [19]. MicroRNA-101 is downregulated in bladder transitional cell carcinoma (TCC) tissues and inhibits cell proliferation and colony formation in TCC cell lines by directly repressing oncogene EZH2 [20]. A recent study indicated that miRNA-let-7a inhibits the expression of MYC and reverses MYC-induced growth in Burkitt lymphoma cells [21]. Previous reports indicate that let-7 is poorly expressed in a variety of human tumors and reduced let-7 level results in over-expression (cyclinD, RAS, MYC) of let-7-responsive genes in tumors [22], [23], [24], [25]. However, the exact role of let-7 in cancer is not yet fully understood. Our preliminary data show that let-7b is down-regulated in human lung squamous tumors, while levels of CYP2J2 protein is up-regulated, suggesting that human CYP2J2 might be post-transcriptionally regulated by let-7b. Hence, the purpose of the present study was to investigate this hypothesis that let-7b might act as a tumor suppressor through targeting CYP2J2.

## Results

### Let-7b Targets the 3′UTR of *CYP2J2*

**Figure 1:**
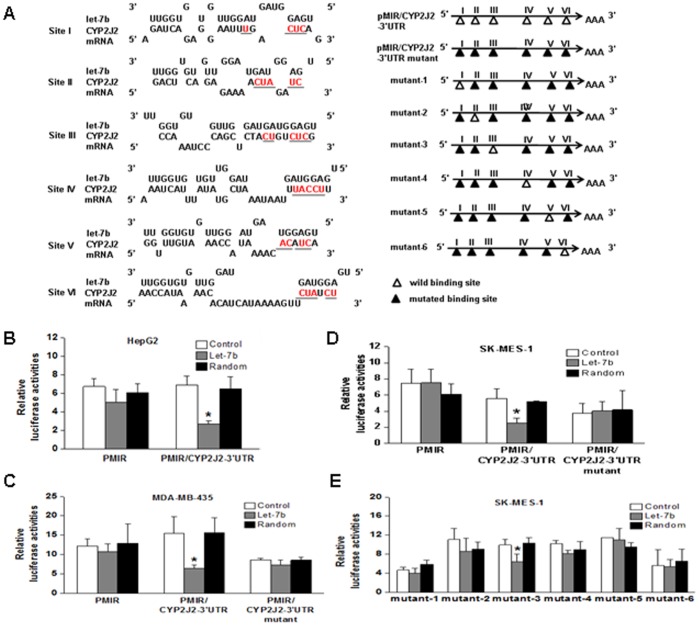
Identification of CYP2J2 as a direct target of let-7b. **A**, schematic representation of the predicted target sites of let-7b in the 3′UTR of CYP2J2. The 3′UTR of CYP2J2 was cloned into a luciferase reporter plasmid, termed pMIR/CYP2J2-3′UTR. A series of mutants carried mutated nucleotides in six potential binding sites for the let-7b seed region were generated based on wild type pMIR/CYP2J2-3′UTR. Mutants of pMIR/CYP2J2-3′UTR are constructed by mutating the complementary site (labeled by underline) in the let-7b seed region to their complementary bases. **B**, luciferase activity was analyzed in HepG2 cells 24 h after transfection with reporter plasmid pMIR/CYP2J2-3′UTR or pMIR (empty vector). **C–D**, full-length sequence CYP2J2-3′UTR containing mutant binding sites for the let-7b seed region were generated based on wild type pMIR/CYP2J2-3′UTR. We transfected these constructs into the cells (MDA-MB-435 and SK-MES-1) and analysed luciferase reporter activity. As we expected, let-7b did not affect luciferase activity of mutants compared with wild type. **E**, the six mutants were transfected into SK-MES-1 cells, in addition to let-7b or random let-7b. The luciferase activity of the six mutants was not repressed by let-7b. Renilla luciferase activities were used to normalize firefly luciferase activity. Columns, mean of three experiments; bars, SD. *, *P*<0.05.

To investigate if CYP2J2 is regulated directly by let-7b, we constructed a luciferase reporter plasmid containing the 3′UTR of CYP2J2 cloned downstream of the luciferase reporter gene (Figure 1A). We transfected the luciferase construct into HepG2 cells together with let-7b or random let-7b. We found that transfection of pMIR/CYP2J2-3′UTR along with let-7b resulted in a significant reduction in reporter activity than did those of control and random transfections. On the other hand, no significant downregulation of the pMIR reporter activity could be determined when we transfected the pMIR reporter (empty vector) along with let-7b or random let-7b into HepG2 cells (Figure 1B). To further confirm that CYP2J2 is a target of let-7b, we constructed seven mutants (pMIR/CYP2J2-3?UTR mutant, mutant-1, mutant-2 … and mutant-6, respectively) based on pMIR/CYP2J2-3?UTR. Then we transfected these constructs into the cells (MDA-MB-435 and SK-MES-1) and analyzed luciferase reporter activity. The assays showed that luciferase activity of pMIR/CYP2J2-3?UTR mutant was not repressed by let-7b, compared with the wild type pMIR/CYP2J2-3?UTR (Figure 1C and D). Among the remaining six mutants (mutant-1, mutant-2…mutant-6), luciferase activity of mutant-3 was repressed by let-7b, comparing with random and control (*P*<0.05) (Figure 1E). Furthermore, binding site III completely matched the seed sequence of let-7b if wobble base pairing was allowed. These data indicate that CYP2J2 is one of the targets of let-7b.

### Let-7b Inhibits Expression Levels of Endogenous CYP2J2 and its Enzymatic Activity in vitro

**Figure 2:**
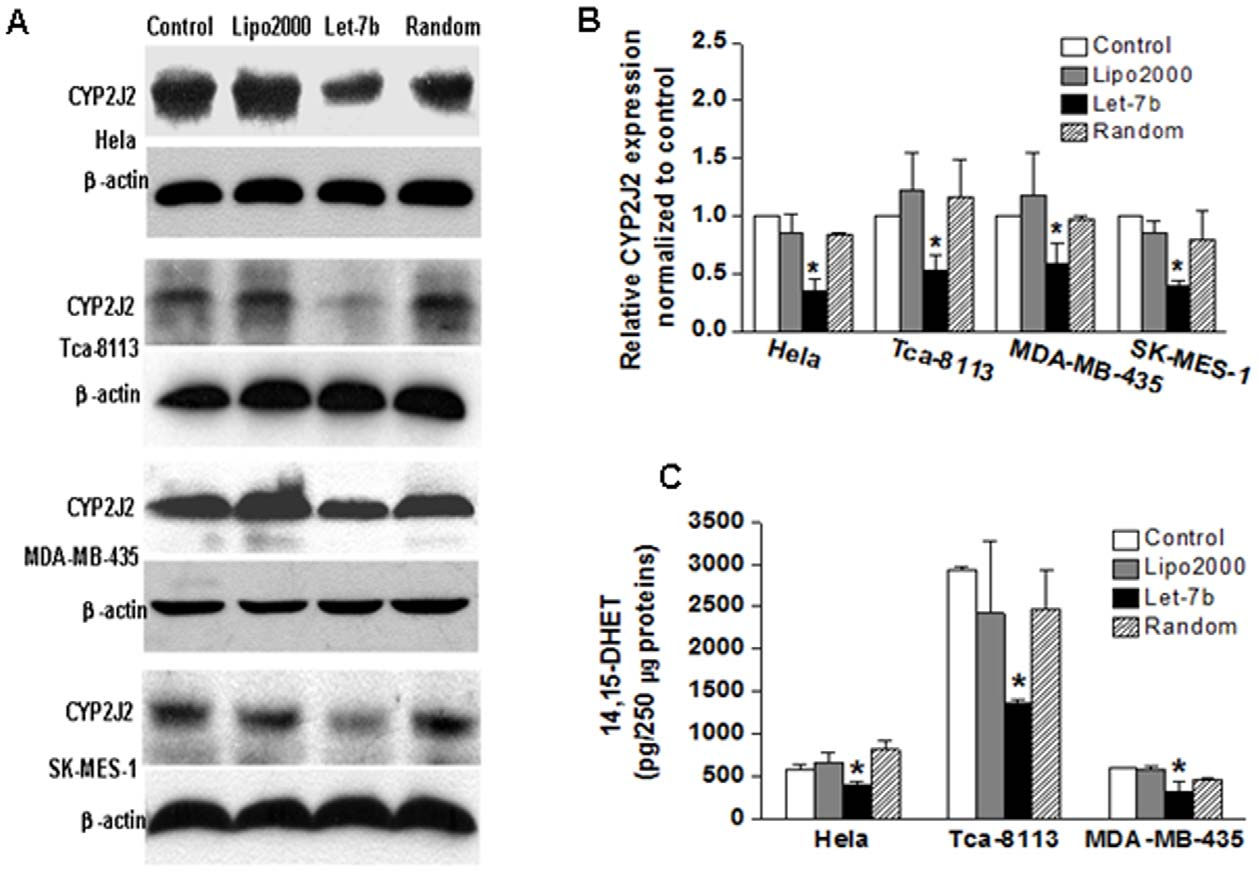
Effect of exogenous let-7b on CYP2J2 expression and its enzymatic activity. **A**, protein level of CYP2J2. HeLa, Tca-8113, MDA-MB-435, and SK-MES-1 cells were treated with let-7b or random let-7b (100 nM) for 48 h. The protein level of CYP2J2 was examined by western blot analysis. **B**, protein level of CYP2J2 was quantified by densitometry. Columns, mean of three experiments; bars, SD. *, *P*<0.05. **C**, stable metabolite 14, 15-DHET in HeLa, Tca-8113, and MDA-MB-435 cells were determined as described under Materials and Methods. Cells treated with exogenous hsa-let-7b produced fewer EETs than those treated with random let-7b. Points, mean of three experiments; bars, SD. *, *P*<0.05.

To investigate the effects of exogenous let-7b on the protein level and enzymatic activity of endogenous CYP2J2 proteins, we examined the protein levels of CYP2J2 in HeLa, Tca-8113, SK-MES-1, and MDA-MB-435 cells after treatment with let-7b for 48 h (100 nM). Western blot analysis showed that overexpression of let-7b significantly downregulated the expression of CYP2J2 in four cancer cell lines (Figure 2A). Relative CYP2J2 expression was quantified by densitometry (Figure 2B). As in Figure S1A and B, western blots showed dose-dependent effect of let-7b and let-7b inhibitor on CYP2J2 protein expression. Given the instability of EETs, we determined concentration of the stable metabolite 14,15-DHET in cell culture media to confirm inhibition of let-7b on the enzymatic activity of CYP2J2. The results showed that 14,15-DHET levels were significantly decreased by the transfection of let-7b (Figure 2C). These data showed that human CYP2J2 is directly downregulated by let-7b.

### Let-7b Reduces Cancer Cell Growth and Induces Apoptosis by Directly Downregulating CYP2J2

**Figure 3:**
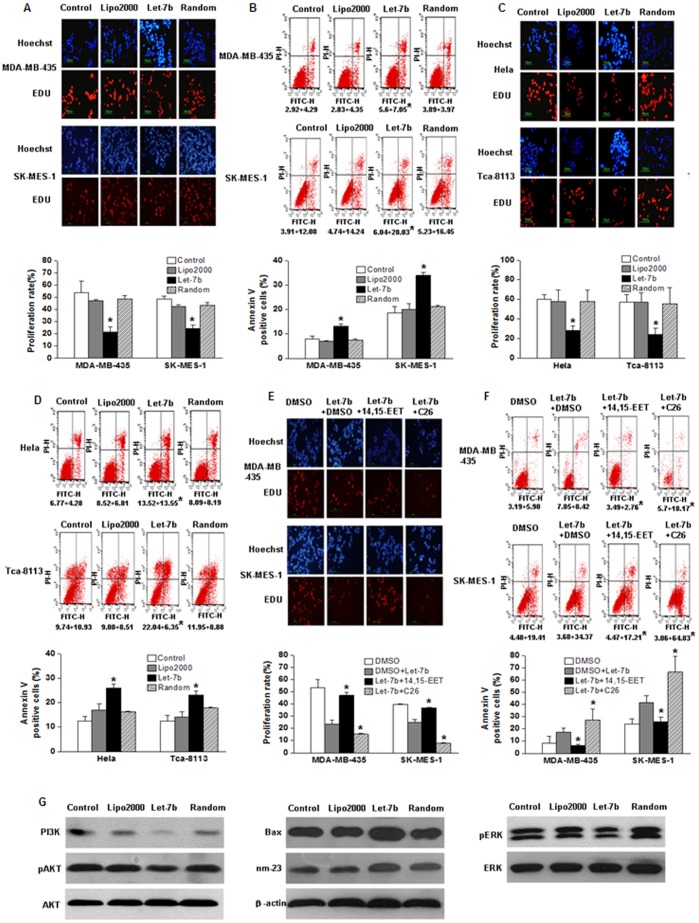
Influence of let-7b on cell proliferation and apoptosis. **A**, proliferation assay via Cell-Light™ EdU DNA Cell Proliferation Kit showing decreased proliferation rate of MDA-MB-435 and SK-MES-1 cells treated with let-7b compared to the cells treated with random let-7b or control. The blue-stained cells were stained by Hoechst, and the red were EdU add-in cells. EdU-positive cells were calculated as (EdU add-in cells/Hoechst-stained cells)×100%. Columns, mean of three experiments; bars, SD. *, *P*<0.05. **B**, percentage of apoptotic cells was increased in MDA-MB-435 and SK-MES-1 cells treated with let-7b. Apoptosis measured by annexin V-FITC (x-axis) and propidium iodide staining (y-axis). Percentages of apoptotic cells (percentage of cells in the upper-right quadrant (annexin V-positive, PI-negative) plus cells in the low-right quadrant (annexin V-positive, PI-positive) in total cell number) are given under the relevant graph. Columns, mean of three experiments; bars, SD. *, *P*<0.05. **C–D**, percentages of EdU-positive cells and apoptotic cells were analyzed in HeLa and Tca-8113 cells transfected with let-7b or random let-7b. **E–F**, MDA-MB-435 and SK-MES-1 cells transfected with let-7b were treated with C26 (specific CYP2J2 inhibitor, 10 µM) or 14,15-EET (250 nM). 48 h later, percentages of EdU-positive cells and apoptotic cells were analyzed. Columns, mean of three experiments; bars, SD. *, *P*<0.05. **G**, let-7b overexpression influenced expression of tumor-related genes in MDA-MB-35 cells. Let-7b overexpression in MDA-MB-435 cells significantly downregulated PI3K, pAkt, and pERK but increased Bax and nm-23 expression. The data shown were repeated three times.

We have previously reported that CYP2J2 stimulates proliferation of carcinoma cells and protects human carcinoma cells from apoptosis [10]; so it was of interest to evaluate the effects of overexpression of let-7b on cell proliferation and apoptosis when endogenous CYP2J2 was inhibited by let-7b. We treated HeLa, Tca-8113, SK-MES-1, and MDA-MB-435 cells with exogenous let-7b or random let-7b for 48 h. The proliferation rate was determined via Cell-Light™ EDU DNA Cell Proliferation Kit (Ribobio, China). The results showed a significant inhibition of cell proliferation by let-7b. For example, in MDA-MB-435 and SK-MES-1 cells, let-7b reduced cell proliferation by 50% compared with negative control and random let-7b (Figure 3A). Apoptosis, measured by Annexin V and propidium iodide staining, significantly increased in cells transfected with let-7b (Figure 3B). In addition, exogenous let-7b decreased the percentage of EDU-positive cells and increased the apoptotic cells in HeLa and Tca-8113 cells (Figure 3C and D). To provide further evidence that the effects of let-7b expression on cell proliferation and apoptosis were related to CYP2J2, cells transfected with let-7b were treated with C26 (10 µM) (specific CYP2J2 inhibitor [12] ) and 14,15-EET (250 nM) for 48 h. To minimize reduction in levels of 14,15-EET due to autooxidation, cells were stimulated with 14,15-EET or a comparable volume of vehicle (DMSO) every 6 h. As expected, the effects of let-7b expression on cell proliferation and apoptosis were enhanced by C26 and abolished by 14,15-EET (Figure 3E and F). In addition, to determine whether let-7b treatment affect cell growth of H9c2 (H9c2 cells do not have *CYP2J2*), we used Cell-Light™ EDU DNA Cell Proliferation Kit and Annexin V and propidium iodide staining to measure the cell proliferation and apoptosis of H9c2 cells. As shown in Figure S2A and B, not only cell proliferation but also cell apoptosis of H9c2 cells weren’t affected by let-7b or let-7b inhibitor treatment. These data suggested that overexpression of let-7b resulted in decreased proliferation and activated apoptosis of carcinoma cell lines.

We also investigated the influences of let-7b on the expression of proapoptotic protein Bax and antimetastatic protein nm-23 and the activation of the PI3K/Akt and MAPK signaling pathways, which play important roles in P450 epoxygenase- and EET-mediated tumorigenesis and metastasis [10], [11], [12]. We found that let-7b overexpression in MDA-MB-435 cells significantly downregulated the PI3K, pAkt, pERK pathways but increased Bax and nm-23 expression (Figure 3G). Similar results were also observed in MDA-MB-435 cell which treated with let-7b agomir (Figure S1C and D). However, let-7b did not affect PI3K, pAkt, and Bax of H9c2 cells (Figure S2C). We speculated that this is because H9c2 cells do not have *CYP2J2*. These results indicated that the enhancing effect of CYP2J2 on tumor formation could be attenuated by let-7b.

### Expression Level of CYP2J2 Protein and let-7b in Human Lung Cancer and in Adjacent Normal Tissues are Inversely Correlated

**Figure 4:**
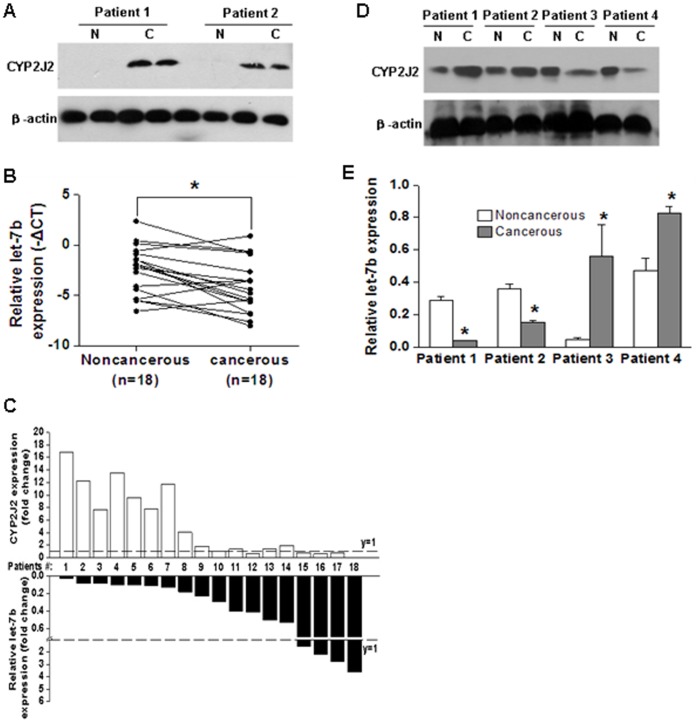
Relationship between CYP2J2 protein and let-7b expression in lung cancer and adjacent nontumor tissues. **A**, expression levels of CYP2J2 in lung cancerous (C) and adjacent nontumor tissues (N) was measured by Western blot analysis. **B**, comparison between the expression levels of let-7b in lung cancerous and adjacent nontumor tissues (n = 18). Although changes of −ΔΔCT values [−(ΔCT_non-tumor tissue_−ΔCT_cancer tissue_)] are relatively mild (from -1.85 to 5.4, 2.6±1.27), the fold changes of let-7b expression between 18 paired human lung squamous cancer and adjacent nontumor tissues are significant (from 0.277 to 42.22, 6.06±2.43). **C**, relationship between CYP2J2 protein and let-7b expression in lung cancer and paired adjacent nontumor tissues. Expression level of CYP2J2 protein was increased in 13 of 18 sets of lung cancer compared with the adjacent normal tissues (the fold change>1). As expected, let-7b levels were commonly reduced in these tumors compared with the adjacent normal tissues (the fold change<1). **D–E**, expression levels of CYP2J2 and let-7b in breast cancerous and adjacent nontumor tissues (n = 4). Real-time RT-PCR was used to determine mature let-7b levels. The U6 snRNA expression level was used to normalize the relative let-7b level. Columns, mean of three experiments; bars, SD. *, *P*<0.05.

To investigate an association between let-7b level and the expression of CYP2J2 in human carcinomas, the expression of CYP2J2 protein and let-7b in 18 paired human lung squamous cancer tissues and adjacent nontumor tissues was examined by western blot analysis and real-time RT-PCR, respectively. Results showed that CYP2J2 was highly expressed in the majority of cancer tissue samples compared with adjacent normal tissues (Figure 4A). Real-time RT-PCR was used to determine the mature let-7b levels in 18 paired human lung squamous cancer tissues and adjacent nontumor tissues. Although changes of −ΔΔCT values [−(ΔCT_non-tumor tissue_−ΔCT_cancer tissue_)] were relatively mild (from −1.85 to 5.4, 2.6±1.27), the fold change (2^−ΔΔCT^) of let-7b expression between 18 paired human lung squamous cancer tissues and adjacent nontumor tissues was significant (from 0.277 to 42.22, 6.06±2.43). Consequently, the results showed that levels of mature let-7b were significantly decreased in 14 lung tumors compared with their matched controls among 18 samples analyzed (Figure 4B). We next investigated a correlation between let-7b expression level and CYP2J2 protein level in human lung squamous cancer tissues. And there is statistically significant inverse correlation between let-7b expression level and CYP2J2 protein level in 18 sets of lung squamous tumors cancer and paired adjacent nontumor tissues (Figure 4C). Furthermore, we found that CYP2J2 had inverse expression levels to let-7b in four paired human breast cancer and adjacent nontumor tissues (Figure 4D and E). And let-7b expression in SK-MES-1 and MDA-MB-435 cells was shown in Figure S3.

### Let-7b-mediated Knockdown of CYP2J2 Inhibits Tumor Growth and Metastasis

**Figure 5:**
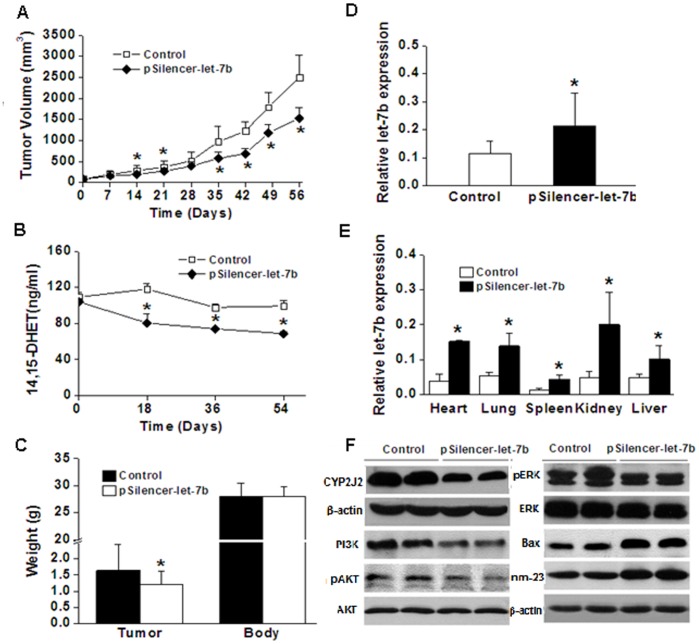
Effect of let-7b on tumor growth. MDA-MB-435 cells were injected subcutaneously into the right flank of nude mice to generate the mouse model of breast cancer. Two weeks later, mice received the let-7b treatment randomly. The let-7b expression vector (pSilencer-let-7b plasmid) was injected into mice through a tail vein at a dose of 4 mg/kg body weight every 3 weeks. **A**, the x-axis was labeled as the days of pSilencer-let-7b treatment. Tumor volume was measured weekly and calculated as TV (mm^3^)  =  length×width^2^×0.5236. **B**, the measurement of 14,15-DHET level in nude mice urine was performed by ELISA according to the manufacturer’s instructions. Points, mean of three experiments; bars, SD. *, *P*<0.05. **C**, average tumor weight and body weight of control and let-7b treatment groups after growth for 8 weeks. Columns, mean; bars, SD. *, *P*<0.05 versus control. **D–E**, the expression of the mature let-7b in the tumors and primary organs was validated by real-time RT-PCR. Columns, mean of three experiments; bars, SD. *, *P*<0.05. **F**, Western blot analysis showed alteration of the expression level of CYP2J2 protein and tumor-related genes of tumor samples.

Furthermore, we investigated the influence of let-7b on tumor growth in an in vivo model. To generate a tumor xenograft model, 2×10^6^ MDA-MB-435 cells were injected subcutaneously into the right flank of nude mice. Two weeks after injection, when tumors had grown to approximately 40 mm^3^, the let-7b expression vector pSilencer-let-7b was injected into mice at a dose of 4 mg/kg body weight through tail vein every 3 weeks. As expected, mice injected with pSilencer-let-7b via tail vein showed a significant reduction in tumor volume compared to the controls (Figure 5A). During the treatment period, we determined the 14,15-DHET level in the urine of nude mice and found a significant reduction in 14,15-DHET in the let-7b treatment group compared with the controls (Figure 5B). At the end of the treatment period, let-7b resulted in significantly decreased tumor weight, but body weight had not changed (Figure 5C). We also treated MDA-MB-435 cell with let-7b agomir (150 nM) or agomir control for 48 h, and then injected these cells into the right flank of nude mice. After 4 weeks, necropsies were performed, and all tumors per mouse were weighed. Let-7b agomir resulted in significantly decreased tumor weight (Figure S4). Furthermore, the level of mature let-7b was validated by real-time RT-PCR; results showed that pSilencer-let-7b treatment resulted in a significant increase in both tumor and organs (Figure 5D and E). Western blot analysis showed a markedly decreased expression level of CYP2J2 protein and a significantly increased expression level of proapoptotic protein Bax and antimetastatic protein nm-23 in the let-7b-treated mice compared with controls (Figure 5F). These results suggest that let-7b can inhibit the expression and tumor-promoting functions of CYP2J2.

**Figure 6:**
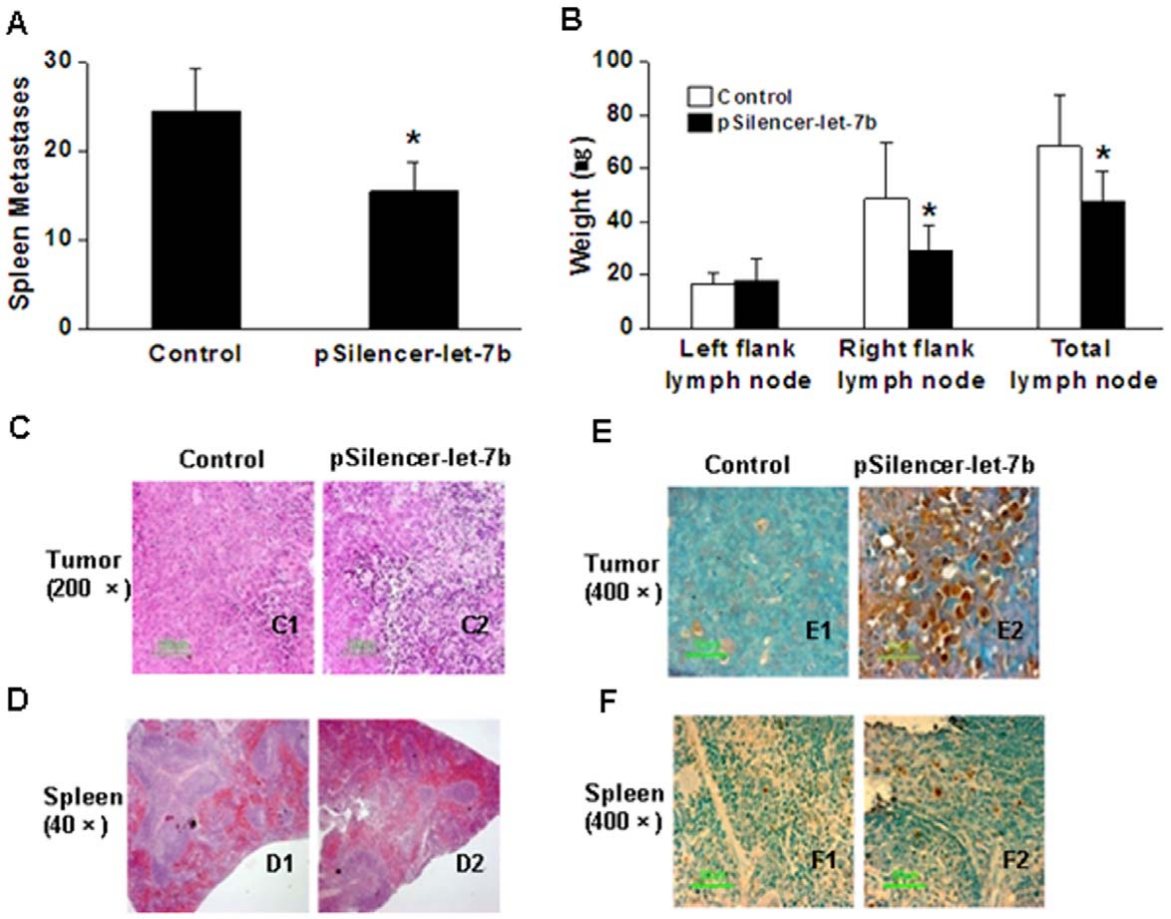
Let-7b overexpression inhibits tumor metastasis. **A**, average number of spleen metastases for each group (n = 6). Columns, mean; bars, SD. *, *P*<0.05 versus control. **B**, average weight of axillary lymph nodes for each group. Columns, mean; bars, SD. *, *P*<0.05 versus control. **C–D**, hematoxylin and eosin staining of sections of tumor (C1, C2) and spleen (D1, D2). **E–F**, TUNEL staining of tumor (E1, E2) and spleen (F1, F2) sections.

Furthermore, we evaluated whether overexpression of let-7b affected metastasis. At the end of the experiment, spleens were removed and incised vertically to count metastatic tumor colonies. Metastases in longitudinal section of the spleen were visible to the naked eye as nodules. We observed a significant decrease in the average number of spleen metastases in mice injected with pSilencer-let-7b compared with controls (Figure 6A). We also determined the extent of lymph node metastasis by measuring the weight of lymph nodes. We found that injection of pSilencer-let-7b through tail vein reduced the average weight of axillary lymph nodes (Figure 6B). Further histological analysis after hematoxylin and eosin staining of paraffin sections showed a marked difference between athymic mice treated with let-7b and controls (Figure 6C and D): the let-7b treatment caused a significant increase in the incidence of necrotic regions in tumor and spleen. Compared with the control group, the metastases focis were smaller and fewer in sections of spleen of the let-7b treatment group. Furthermore, TUNEL staining of sections showed that let-7b treatment resulted in a significant increase in TUNEL-positive cells in tumors and in mice spleen (Figure 6E and F). These data indicate that let-7b treatment can inhibit tumor metastasis.

## Discussion

In the present study, we have shown a new mechanism for controlling the tumor-promotion function of CYP2J2. Bioinformatics analysis predicted that let-7b is a potential regulator of *CYP2J2* gene. Using CYP2J2-expression cancer cell lines and a tumor xenografs model, we have shown that human *CYP2J2* is posttranscriptionally regulated by let-7b and that the posttranscriptional regulation is responsible for the tumor-promotion function of CYP2J2. Furthermore, in human lung squamous cancer and adjacent nontumor tissues, we observed a reverse relationship between let-7b and CYP2J2 expression levels.

It has become apparent that microRNAs regulate the expression of target genes by binding to the complementary regions of the 3′UTR of their target genes [26], but whether hsa-let-7b regulates human CYP2J2 expression is not known. Previous study has shown that let-7 regulates RAS through its 3′UTR [27]. In the present study, luciferase assays showed that let-7b repressed the activity of the reporter construct containing the 3′UTR region of CYP2J2 mRNA. Transfection of carcinoma cell lines with let-7b and injection of pSilencer-let-7b producing mature let-7b in vivo decreased CYP2J2 protein expression and enzymatic activity, suggesting that *CYP2J2* is posttranscriptionally negatively regulated by let-7b.

Recent studies have demonstrated that let-7 might act as a tumor suppressor and that reduced let-7 level results in let-7-responsive gene (cyclinD, RAS, MYC, etc.) overexpression in tumors [22], [23], [24], [25]. With western blot analysis and real-time RT-PCR, we showed higher expression of CYP2J2 protein and lower expression of let-7b in 18 paired human lung squamous cancer tissues compared with the adjacent nontumor tissues. Thus, the high expression of CYP2J2 protein in cancer tissues may result from the decreased expression of let-7b, at least in part. Of course, it is likely that another mechanism(s) is involved in regulating CYP2J2 expression. Our previous study showed that overexpression of CYP2J2 or additing exogenous EETs decreased apoptosis and increased cell proliferation in cancer cell lines and increased tumor growth and lung metastasis in a murine xenograft model [10]. Furthermore, we recently found that Compound 26, the selective inhibitor of CYP2J2, significantly repressed the tumor-promotion function of CYP2J2 [12].

In the present study, we described a new mechanism for suppression of CYP2J2. We treated carcinoma cell lines with let-7b and found that overexpression of let-7b resulted in decreased proliferation and activated apoptosis of cancer cells. We also found that pSilencer-let-7b treatment significantly inhibited tumor growth in a tumor xenograft model. Although downregulation of CYP2J2 by pSilencer-let-7b did not result in the eradication of tumors, the reverse correlation between the expression level of CYP2J2 and let-7b in human cancer tissue and the effectiveness of inhibiting the tumor-promoting functions of CYP2J2 revealed the potential therapeutic benefit of let-7b in human cancers.

In our previous study, we reported that EETs protect endothelial cells from apoptosis by activating the phosphatidylinositol 3-kinase (PI3K)/Akt and MAPK signaling pathways [25]. In the current study, overexpression of let-7b suppressed the expression of CYP2J2 protein and its EET products in vitro and in vivo. We also found that let-7b could inhibit the proliferation, and enhance the apoptosis, of human tumor cells by inhibiting the PI3K/Akt and MAPK signaling pathways and by activating the proapoptotic protein Bax and antimetastatic protein nm-23, both of which are involved in P450 epoxygenase- and EET-mediated tumorigenesis and metastasis [10]. Our data suggest that the effect of CYP2J2-derived EETs on tumor formation was mediated by let-7b. The 11 members of the let-7 family have similar target genes and functions on cell proliferation because of the high similarity among their sequences [23], [28]. In the present study, we focused on the effect of let-7b on the expression of CYP2J2. Effects of the other members of let-7 family on CYP2J2 expression and cancer cell proliferation need further study.

In conclusion, our data confirmed that let-7b expression is frequently decreased in lung squamous cell cancers. Our work also indicated that human CYP2J2 is posttranscriptionally regulated by let-7b, which results in the high expression of CYP2J2 protein in human carcinomas. Our study demonstrated a new mechanism whereby CYP2J2- and EET-mediated tumorigenesis and metastasis are associated with let-7b. Besides MYC and RAS, let-7b can function as a tumor suppressor by blocking CYP2J2.

## Materials and Methods

### Ethics Statement

This study was approved by the Review Board of Tongji Hospital and Tongji Medical College. The recruited subjects provided written informed consent. The investigation conforms to the principles outlined in the Declaration of Helsinki. All animal experimental protocols complied with the “Guide for the Care and Use of Laboratory Animals” published by the United States National Institutes of Health.

### Cell Lines

The human cervix adenocarcinoma cell line HeLa, human hepatoma liver cell line HepG2, human tongue squamous cell carcinoma Tca-8113, and human lung squamous cancer cell line SK-MES-1 were obtained from the American Type Culture Collection (Manassas, Virginia). Cells were maintained in Dulbecco’s modified Eagle’s medium (DMEM) (Invitrogen, Carlsbad, California) with 10% fetal bovine serum (FBS) at 37°C in the presence of 5% CO_2_ at constant humidity. The MDA-MB-435 human breast carcinoma cell line was also obtained from the American Type Culture Collection and was grown in RPMI 1640 medium supplemented with 10% FBS and maintained at 37°C in 95% air/5% CO_2_.

### MicroRNA Target Prediction

RNAhybrid (http://bibiserv.techfak.uni-bielefeld.de/cgi-bin/rnahybrid_submit) was used for miRNA target prediction. We found four potential target sites (site I, site III, site IV, and site VI) in 3′UTR of human CYP2J2 for let-7b. In addition, we also compared the let-7b sequence to the full length sequence of CYP2J2-3′UTR with DNAMAN software (LynnonBiosoft, Quebec, Canada). We also found another two potential bingding sites (site II and site V), besides the four potential target sites previously described (Figure 1A).

### Plasmid Construction

The full length sequence of human CYP2J2-3?UTR was amplified by PCR using the primers 5′-GCGACTAGTGTATCACCATTTCCCCAGTCAGTCA-3′ (CYP2J2-3′UTR-former primer) and 5′-GCGAAGCTTCATGGGAATAAGTGTCTGATGGAGG-3′ (CYP2J2-3′UTR-reverse primer). To construct luciferase reporter plasmids, the 3′UTR sequence of CYP2J2 was inserted into the HindIII and SacI sites, downstream of the luciferase reporter gene in the pMIR-REPORT™ Luciferase Vector (Ambion, Carlsbad, California). This plasmid was termed pMIR/CYP2J2-3′UTR. Correspondingly, constructs containing CYP2J2-3′UTR with point mutations in the seed sequence were constructed using the Fast Mutagenesis System kit (TransGen, Biotech, China) according to the manufacturer’s protocol. PMIR/CYP2J2-3′UTR mutant, which carried mutated sequence in the complementary site for the let-7b seed region in all the six potential binding sites, was generated based on the wild type plasmid pMIR/CYP2J2-3′UTR. Furthermore, the other six plasmids, in which one of the six potential binding sites was wild and the other five putative binding sites were mutated, were termed mutant-1, mutant-2, mutant-3, mutant-4, mutant-5, and mutant-6, respectively. For example, the construct, in which the seed-matching sequence of site I was not mutated and the other five were mutated, was named mutant-1. Mutant-2 carried the mutated seed-matching sequence of five binding sites except site II. The remaining mutants were named by analogy (Figure 1A).

To construct the let-7b expression vector (pSilencer-let-7b), two complementary single oligonucleotide strands, 5′-GATCCAACCACACAACCTACTACCTCATTCAAGAGATGAGGTAGTAGGTTGTGTGGTTA-3′UTR and 5′-AGCTTAACCACACAACCTACTACCTCATCTCTTGAATGAGGTAGTAGGTTGTGTGGTTG-3′UTR, were synthesized (AuGCT, China). After an initial denaturation at 94°C for 3 min, the two complementary oligonucleotides were incubated at 37°C for 1 h to get double-strand DNA fragments. The annealed DNA fragments were ligated into BamHI/HindIII sites of plasmid pSilencer 4.1-CMV vector (Ambion, Carlsbad, California). This pSilencer-let-7b plasmid expresses a let-7b RNA hairpin that yields mature let-7b.

### Luciferase Assay

HepG2, MDA-MB-435, and SK-MES-1 cells were transfected in 24-well plates by Lipofectamine 2000 (lipo2000) (Invitrogen, Carlsbad, California) in accordance with the manufacturer’s instructions. Cells were transiently transfected with 200 ng of the luciferase constructs pMIR/CYP2J2-3′UTR or the empty vector and 10 ng of pRL-TK plasmid (Promega, Madison, Wisconsin). In some cases, let-7b (mature let-7b) or random let-7b (Ribobio, China) was cotransfected with reporter plasmids in a final concentration of 100 nM. Random let-7b was the scrambled oligonucleotide and used as negative control. Luciferase activity was analyzed 24 h after transfection by using the Dual-Luciferase Reporter Assay System (Promega, Madison, Wisconsin). Renilla luciferase activities were used to normalize the transfection efficiency and the internally controlled firefly luciferase activity.

### Western Blotting

Cells grown in six-well plates were transfected with let-7b or random let-7b to a final concentration of 100 nM. 48 h after transfection, cells were harvested and homogenized with lysis solution (50 mM Tris-Cl, pH 8.0; 150 mM NaCl; 0.02% sodium azide; 0.1% SDS; 1 µg/ml aprotinin; 1% Nonidet P-40; and 0.5% sodium deoxycholate) containing protease inhibitors (100 µg/ml phenylmethylsulfonyl fluoride, 2 µg/ml aprotinin, 2 µg/ml leupeptin). Supernatant was collected after centrifuging at 12,000×g for 20 min at 4°C. The protein concentration was measured using the BCA protein assay reagent kit (Boster, China). Lysates were resolved by 10% SDS-polyacrylamide gel electrophoresis and transferred to polyvinylidene difluoride (PVDF) membranes. After blocking with 5% nonfat milk, blots were probed with a specific antibody and incubated with a peroxidase-conjugated secondary antibody. Bands were visualized by enhanced chemiluminescence reagents (Pierce Chemical, Rockford, Illinois) and quantified by densitometry.

### Determining 14,15-dihydroxyeicosatrienoic Acid Levels

Measuring EET and 14,15-DHET in the cultured cells and urine of nude mice was performed as previously described [10], [11], [12].

### Cell Proliferation and Apoptosis Assays

Cell proliferation was monitored using Cell-Light EDU DNA Cell Proliferation Kit (Ribobio, China). Cells grown in 96-well plates were transfected with miRNA as described. Proliferation assays were done 48 h after transfection following the manufacturer’s protocol. The blue-stained cells were stained by Hoechst, and the red were EdU add-in cells. EdU add-in cells and Hoechst-stained cells were counted in six randomly selected microscopic fields per well. EdU-positive cell was calculated as (EdU add-in cells/Hoechst stained cells) ×100%.

To measure the effects on cell apoptosis rates, cells were harvested 48 h posttransfection and resuspended in binding buffer. FITC-conjugated annexin V and propidium iodide (Keygentec, China) were added to the cells, and samples were incubated for 15 min at room temperature. Cells were then analyzed with a FACStar-Plus flow cytometer (BD Biosciences, Franklin Lakes, New Jersey) to determine the percentage of apoptotic cells. We calculated the percentage of apoptotic cells by the percentage of cells in the upper-right quadrant (annexin V-positive, PI-positive) plus cells in the lower right quadrant (annexin V-positive, PI-negative) in the total number of cells.

### RNA Extraction and Real-time PCR

Total RNA was extracted from frozen tissue samples by using Trizol Reagent Kit (Invitrogen, Carlsbad, California) according to the manufacturer’s instructions. Two micrograms of total RNA were reverse-transcribed using a reverse transcription kit (Takara, Japan). Quantification of miRNA was done by quantitative real-time PCR according to the manufacturer’s protocol (Ribobio, China). Expression of U6 small nuclear RNA (snRNA) was used as an internal standard.

The specific primers were purchased from Ribobio Company (Ribobio, China). The PCR profiles for human let-7b and human U6 snRNA were performed as follows: after an initial denaturation at 95°C for 20 s, amplification was finished by denaturing at 95°C for 10 s, annealing at 60°C for 20 s, and extending at 70°C for 1 s through 40 cycles.

### Measuring Let-7b and CYP2J2 Levels in Human Lung Cancerous and Adjacent Noncancerous Tissues

Lung cancerous and adjacent noncancerous tissues were collected during surgical procedures at Tongji Hospital from 18 patients with lung squamous cell cancer. Informed consents were obtained. In addition, breast cancer and adjacent noncancerous tissues were also collected from four patients. The samples were immediately frozen in liquid nitrogen and stored at −80°C. Paired nontumor and tumor tissues were further examined by quantitative real-time reverse transcription-PCR (qRT-PCR) for the expression levels of let-7b and by Western blot analysis for CYP2J2 as described above. This study was approved by the Clinical Research Committee of Tongji Medical College and was carried out according to the guidelines of the NIH.

### Xenograft Model of Tumor Growth

All animal studies were approved by the Animal Research Committee of Tongji Medical College and were carried out in accordance with the guidelines of the NIH (approval ID: S215). Male athymic BALB/C nude mice 4 weeks old were housed in specific pathogen-free conditions on a daily 12-h light/12-h dark cycle. MDA-MB-435 cells were harvested on the day of use and injected subcutaneously into the right flank of the nude mice. Approximately 2 weeks after implantation, when tumors had grown to approximately 40 mm^3^, mice were randomly divided into control and let-7b precursor expression vector treatment groups (n = 16). Mice received either let-7b precursor expression plasmid (pSilencer-let-7b) at a dose of 4 mg/kg body weight or a comparable dose of empty plasmid pSilencer (dissolved in 100 µl deionized water) as control through tail vein injection every 3 weeks. The mice were monitored daily and weighed weekly. Tumor volume was calculated weekly according to the formula (TV (mm^3^)  =  length×width^2^×0.5236). At the end of the experiment, tumor xenografts were harvested, weighed, and snap-frozen. Tissues were fixed in 3.7% formalin, embedded in paraffin, and serially cut into 4-µm thick sections for hematoxylin and eosin staining and TUNEL assay.

### Terminal Deoxynucleotidyl Transferase–mediated dUTP Labeling

Apoptosis in tissue**s** was detected by using the TACS® 2 TdT DAB In Situ Apoptosis Detection Kit according to the manufacturer’s instruction (R&D Systems, Minneapolis, Minnesota). Sections were incubated with 20 µg/ml proteinase K for 15 min and then immersed in 3% H_2_O_2_ for 5 min to quench endogenous peroxidase. After being immersed in 1×TdT Labeling Buffer for 5 min, the sections were incubated in TdT Labeling Reaction Mix for 60 min at 37°C in a humidity chamber and then incubated with 1×TdT Stop Buffer for 5 min to stop the labeling reaction. After deionized water washes, sections were incubated with Strep-HRP solution for 10 min at 37°C, washed twice with 1×PBS, and immersed in DAB solution for 2 to 7 min. Sections were then counterstained with methyl green, and the TUNEL-positive cells were counted in five fields per tissue section.

### Statistical Analysis

Data are presented as mean ± SD from at least three separate experiments. The data were analyzed by Student’s *t*-test. A P value <0.05 was considered as significant for all tests.

## Supporting Information

Figure S1 **Effect of exogenous let-7b on CYP2J2 expression.** **A**, MDA-MB-435 cells were treated with let-7b or let-7b random (50 nM, 100 nM, 150 nM and 200 nM) for 48 hours. The protein level of CYP2J2 was examined by western blot analysis. **B**, MDA-MB-435 cells were treated with let-7b inhibitor or inhibitor control (50 nM, 100 nM, 150 nM and 200 nM) for 48 hours. Expression of CYP2J2 was up-regulated by let-7b inhibitor. **C**, MDA-MB-435 cells were treated with let-7b agomir (150 nM), let-7b antagomir (150 nM) or negative control for 48 h. Expression of CYP2J2 was down-regulated by let-7b agomir and up- regulated by let-7b antagomir. **D**, western blot analysis was used to detect the expression level of PI3K/AKT and BAX/nm-23 in MDA-MB-435 cells treated with let-7b agomir or antagomir.
(TIF)

Figure S2 **Influence of let-7b on cell proliferation and apoptosis in H9C2 cell. A**, proliferation assay via Cell-LightTM EDU DNA Cell Proliferation Kit showing proliferation rate of MDA-MB-435 cell treated with let-7b (150 nM) or let-7b inhibitor (150 nM). Let-7b random and inhibitor control were used as control. Columns, mean of three experiments; bars, SD. **B**, percentage of apoptotic cells was increased in MDA-MB-435 treated with let-7b (150 nM) or let-7b inhibitor (150 nM). Percentages of apoptotic cells (percentage of cells in the upper-right quadrant (annexin V-positive, PI-negative) plus cells in the low-right quadrant (annexin V-positive, PI-positive) in total cell number) are given under the relevant graph. Columns, mean of three experiments; bars, SD. **C**, Influence of let-7b or let-7b inhibitor overexpression on expression of PI3K, pAkt, and pERK and Bax in H9c2 cells. No significant difference of PI3K, pAkt, and pERK and Bax protein expression level were observed in H9c2 cells transfected with let-7b or let-7b inhibitor.
(TIF)

Figure S3 **Expression of let-7b in SK-MES-1 and MDA-MB-435 cells.** Real-time RT-PCR was used to determine mature let-7b levels. U6 served as an internal normalized reference. Columns, mean of three experiments; bars, SD.
(TIF)

Figure S4 **Tumor weight of MDA-MB-435 cells transfected with agomir control or let-7b agomir.** MDA-MB-435 cell were transfected with let-7b agomir (150 nM) or agomir control for 48 h, and then subcutaneously injected into the right flank of nude mice. After 4 weeks, the mice were sacrificed, necropsies were performed, and all tumors per mouse were weighed. Columns, mean; bars, SD. *, *P*<0.05.
(TIF)

## References

[1] Capdevila JH, Falck JR, Harris RC (2000). Cytochrome P450 and arachidonic acid bioactivation: molecular and functional properties of the arachidonate monooxygenase.. Journal of Lipid Research.

[2] Wu S, Moomaw CR, Tomer KB, Falck JR, Zeldin DC (1996). Molecular cloning and expression of CYP2J2, a human cytochrome P450 arachidonic acid epoxygenase highly expressed in heart.. J Biol Chem.

[3] Enayetallah AE, French RA, Thibodeau MS, Grant DF (2004). Distribution of soluble epoxide hydrolase and of cytochrome P450 2C8, 2C9, and 2J2 in human tissues.. J Histochem Cytochem.

[4] Zeldin DC (2001). Epoxygenase pathways of arachidonic acid metabolism.. J Biol Chem.

[5] Karara A, Makita K, Jacobson HR, Falck JR, Guengerich FP (1993). Molecular cloning, expression, and enzymatic characterization of the rat kidney cytochrome P-450 arachidonic acid epoxygenase.. J Biol Chem.

[6] Wu S, Chen W, Murphy E, Gabel S, Tomer KB (1997). Molecular cloning, expression, and functional significance of a cytochrome P450 highly expressed in rat heart myocytes.. J Biol Chem.

[7] Spector AA, Fang X, Snyder GD, Weintraub NL (2004). Epoxyeicosatrienoic acids (EETs): metabolism and biochemical function.. Prog Lipid Res.

[8] Node K, Huo Y, Ruan X, Yang B, Spiecker M (1999). Anti-inflammatory properties of cytochrome P450 epoxygenase-derived eicosanoids.. Science.

[9] Wang Y, Wei X, Xiao X, Hui R, Card JW (2005). Arachidonic acid epoxygenase metabolites stimulate endothelial cell growth and angiogenesis via mitogen-activated protein kinase and phosphatidylinositol 3-kinase/Akt signaling pathways.. J Pharmacol Exp Ther.

[10] Jiang JG, Chen CL, Card JW, Yang S, Chen JX (2005). Cytochrome P450 2J2 promotes the neoplastic phenotype of carcinoma cells and is up-regulated in human tumors.. Cancer Res.

[11] Jiang JG, Ning YG, Chen C, Ma D, Liu ZJ (2007). Cytochrome p450 epoxygenase promotes human cancer metastasis.. Cancer Res.

[12] Chen C, Li G, Liao W, Wu J, Liu L (2009). Selective inhibitors of CYP2J2 related to terfenadine exhibit strong activity against human cancers in vitro and in vivo.. J Pharmacol Exp Ther.

[13] Chen CZ, Li L, Lodish HF, Bartel DP (2004). MicroRNAs modulate hematopoietic lineage differentiation.. Science.

[14] Cheng AM, Byrom MW, Shelton J, Ford LP (2005). Antisense inhibition of human miRNAs and indications for an involvement of miRNA in cell growth and apoptosis.. Nucleic Acids Res.

[15] Kloosterman WP, Plasterk RH (2006). The diverse functions of microRNAs in animal development and disease.. Dev Cell.

[16] Esquela-Kerscher A, Slack FJ (2006). Oncomirs - microRNAs with a role in cancer.. Nat Rev Cancer.

[17] Godlewski J, Nowicki MO, Bronisz A, Williams S, Otsuki A (2008). Targeting of the Bmi-1 oncogene/stem cell renewal factor by microRNA-128 inhibits glioma proliferation and self-renewal.. Cancer Res.

[18] Mott JL (2009). MicroRNAs involved in tumor suppressor and oncogene pathways: implications for hepatobiliary neoplasia.. Hepatology.

[19] Bandi N, Zbinden S, Gugger M, Arnold M, Kocher V (2009). miR-15a and miR-16 are implicated in cell cycle regulation in a Rb-dependent manner and are frequently deleted or down-regulated in non-small cell lung cancer.. Cancer Res.

[20] Friedman JM, Liang G, Liu CC, Wolff EM, Tsai YC (2009). The putative tumor suppressor microRNA-101 modulates the cancer epigenome by repressing the polycomb group protein EZH2.. Cancer Res.

[21] Sampson VB, Rong NH, Han J, Yang Q, Aris V (2007). MicroRNA let-7a down-regulates MYC and reverts MYC-induced growth in Burkitt lymphoma cells.. Cancer Res.

[22] Akao Y, Nakagawa Y, Naoe T (2006). let-7 microRNA functions as a potential growth suppressor in human colon cancer cells.. Biol Pharm Bull.

[23] Johnson CD, Esquela-Kerscher A, Stefani G, Byrom M, Kelnar K (2007). The let-7 microRNA represses cell proliferation pathways in human cells.. Cancer Res.

[24] Schultz J, Lorenz P, Gross G, Ibrahim S, Kunz M (2008). MicroRNA let-7b targets important cell cycle molecules in malignant melanoma cells and interferes with anchorage-independent growth.. Cell Res.

[25] Yang S, Lin L, Chen JX, Lee CR, Seubert JM (2007). Cytochrome P-450 epoxygenases protect endothelial cells from apoptosis induced by tumor necrosis factor-alpha via MAPK and PI3K/Akt signaling pathways.. Am J Physiol Heart Circ Physiol.

[26] Bartel DP (2004). MicroRNAs: genomics, biogenesis, mechanism, and function.. Cell.

[27] Johnson SM, Grosshans H, Shingara J, Byrom M, Jarvis R (2005). RAS is regulated by the let-7 microRNA family.. Cell.

[28] Zhao Y, Deng C, Wang J, Xiao J, Gatalica Z (2011). Let-7 family miRNAs regulate estrogen receptor alpha signaling in estrogen receptor positive breast cancer.. Breast Cancer Res Treat.

