# Supplementary figures and images for: Let-7b Inhibits Human Cancer Phenotype by Targeting Cytochrome P450 Epoxygenase 2J2
Source: PLoS One. 2012 Jun 25;7(6):e39197. doi: 10.1371/journal.pone.0039197 (PMC3382602; doi:10.1371/journal.pone.0039197)

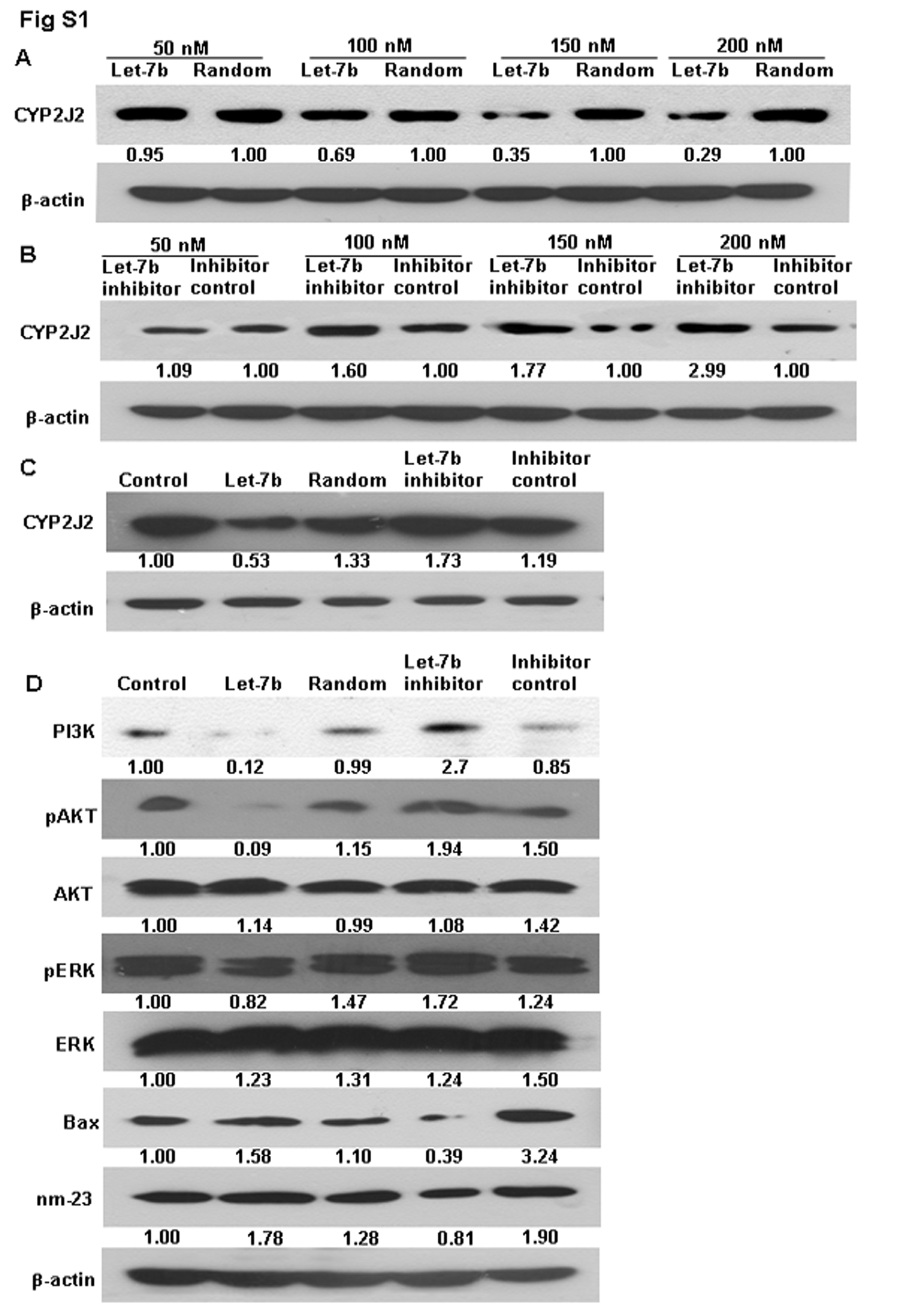

Supplement: Figure S1 — Effect of exogenous let-7b on CYP2J2 expression. A, MDA-MB-435 cells were treated with let-7b or let-7b random (50 nM, 100 nM, 150 nM and 200 nM) for 48 hours. The protein level of CYP2J2 was examined by western blot analysis. B, MDA-MB-435 cells were treated with let-7b inhibitor or inhibitor control (50 nM, 100 nM, 150 nM and 200 nM) for 48 hours. Expression of CYP2J2 was up-regulated by let-7b inhibitor. C, MDA-MB-435 cells were treated with let-7b agomir (150 nM), let-7b antagomir (150 nM) or negative control for 48 h. Expression of CYP2J2 was down-regulated by let-7b agomir and up- regulated by let-7b antagomir. D, western blot analysis was used to detect the expression level of PI3K/AKT and BAX/nm-23 in MDA-MB-435 cells treated with let-7b agomir or antagomir. (TIF) [file pone.0039197.s001.tif]

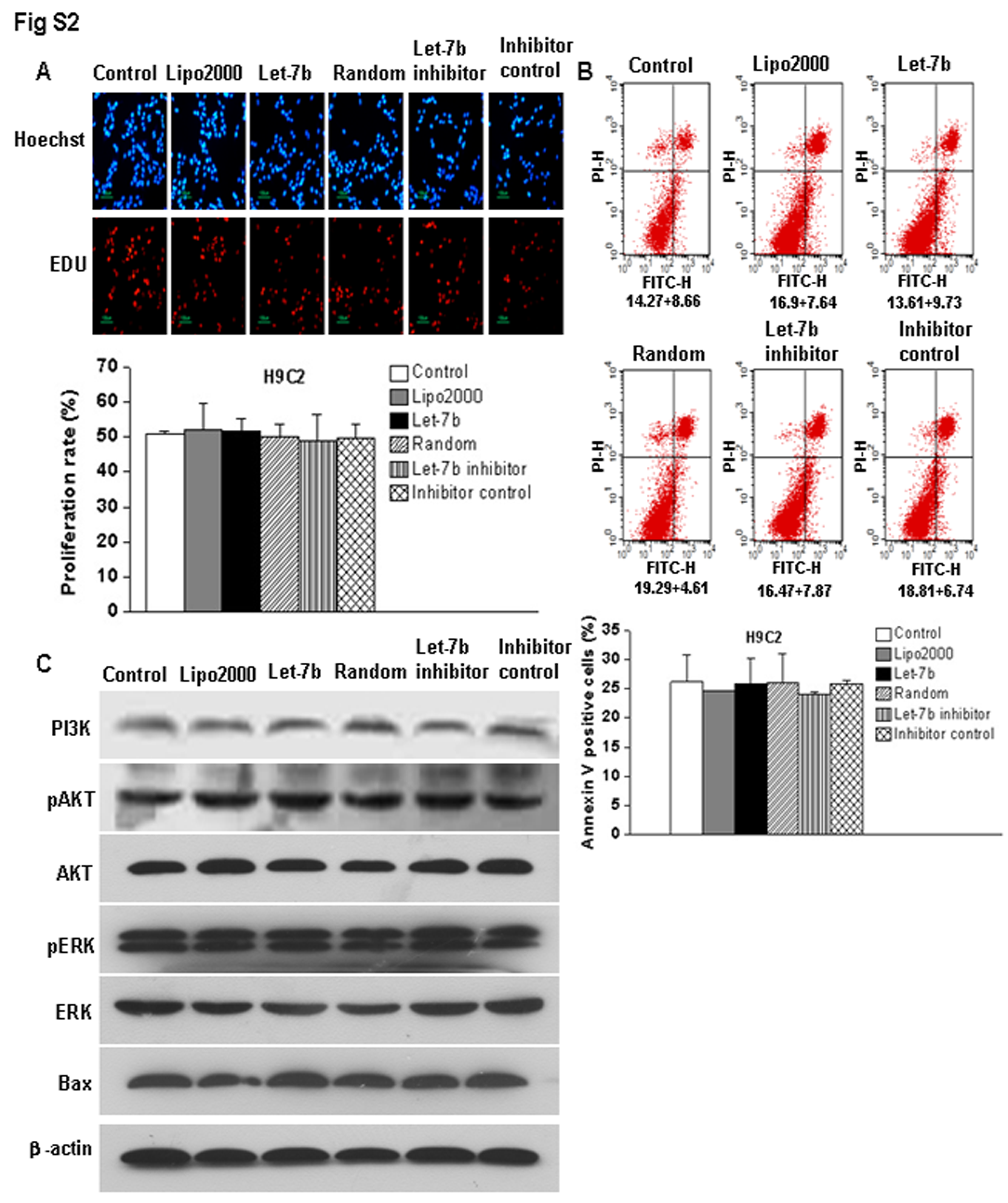

Supplement: Figure S2 — Influence of let-7b on cell proliferation and apoptosis in H9C2 cell. A, proliferation assay via Cell-LightTM EDU DNA Cell Proliferation Kit showing proliferation rate of MDA-MB-435 cell treated with let-7b (150 nM) or let-7b inhibitor (150 nM). Let-7b random and inhibitor control were used as control. Columns, mean of three experiments; bars, SD. B, percentage of apoptotic cells was increased in MDA-MB-435 treated with let-7b (150 nM) or let-7b inhibitor (150 nM). Percentages of apoptotic cells (percentage of cells in the upper-right quadrant (annexin V-positive, PI-negative) plus cells in the low-right quadrant (annexin V-positive, PI-positive) in total cell number) are given under the relevant graph. Columns, mean of three experiments; bars, SD. C, Influence of let-7b or let-7b inhibitor overexpression on expression of PI3K, pAkt, and pERK and Bax in H9c2 cells. No significant difference of PI3K, pAkt, and pERK and Bax protein expression level were observed in H9c2 cells transfected with let-7b or let-7b inhibitor. (TIF) [file pone.0039197.s002.tif]

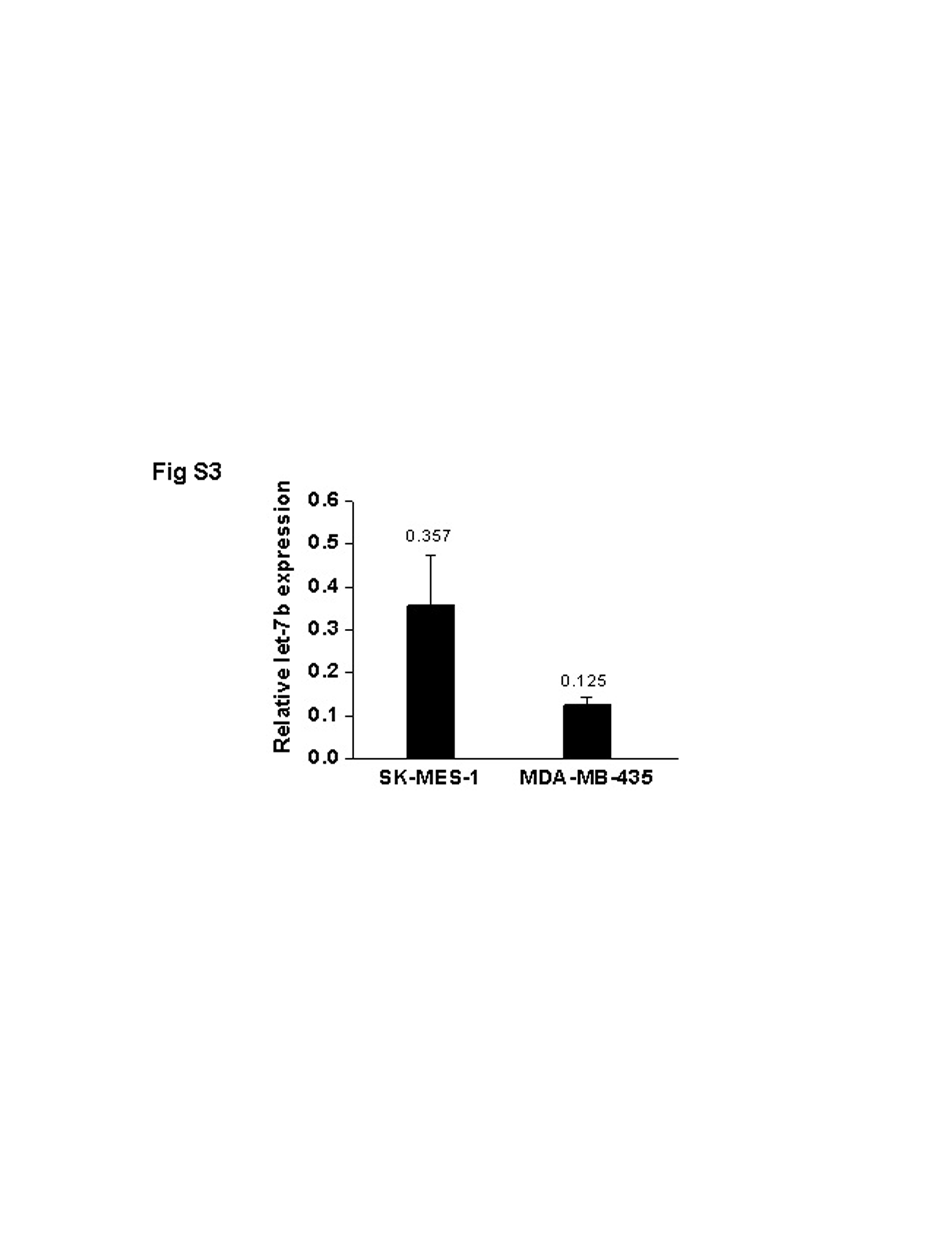

Supplement: Figure S3 — Expression of let-7b in SK-MES-1 and MDA-MB-435 cells. Real-time RT-PCR was used to determine mature let-7b levels. U6 served as an internal normalized reference. Columns, mean of three experiments; bars, SD. (TIF) [file pone.0039197.s003.tif]

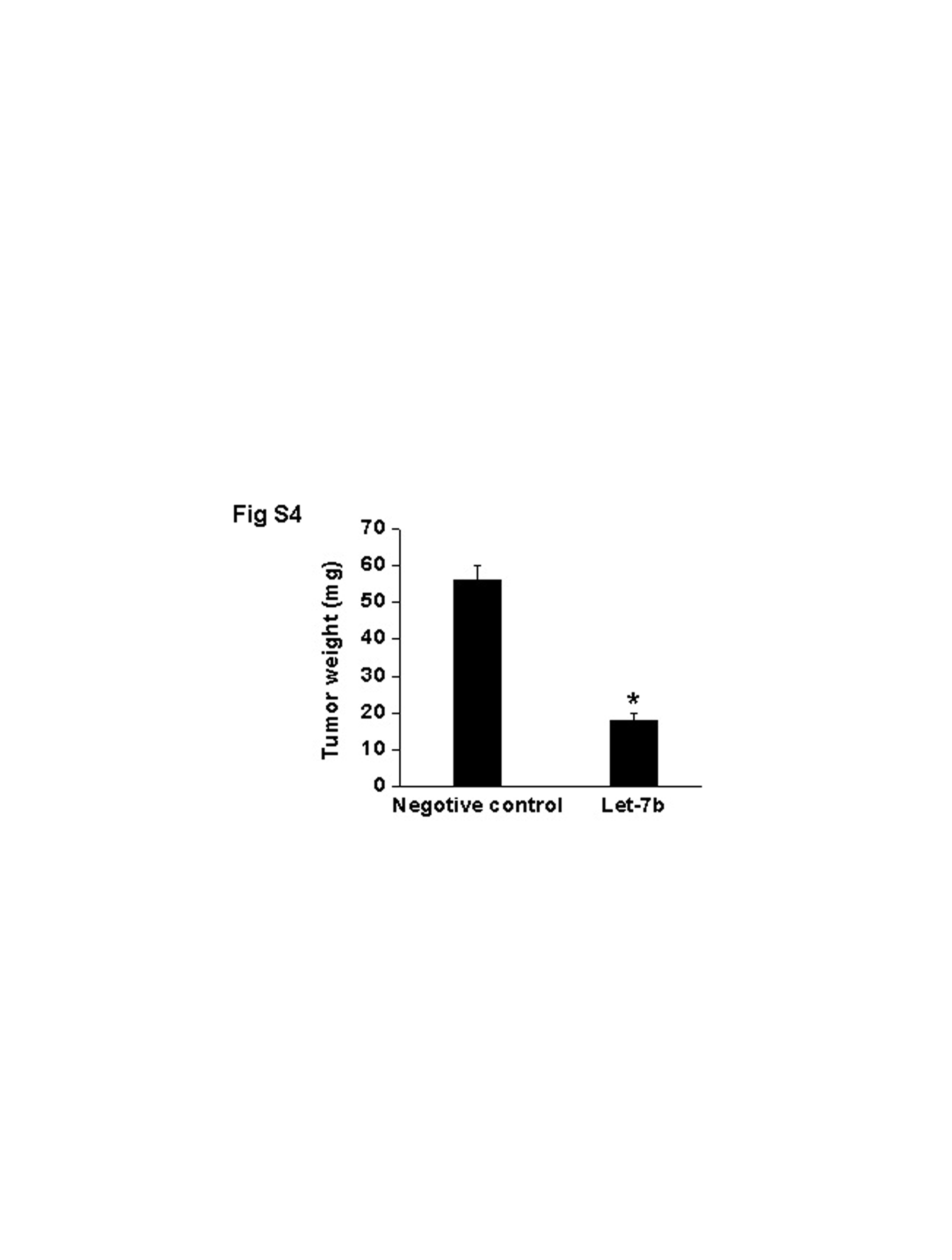

Supplement: Figure S4 — Tumor weight of MDA-MB-435 cells transfected with agomir control or let-7b agomir. MDA-MB-435 cell were transfected with let-7b agomir (150 nM) or agomir control for 48 h, and then subcutaneously injected into the right flank of nude mice. After 4 weeks, the mice were sacrificed, necropsies were performed, and all tumors per mouse were weighed. Columns, mean; bars, SD. *, P<0.05. (TIF) [file pone.0039197.s004.tif]
